# Evolution of mitochondrial TAT translocases illustrates the loss of bacterial protein transport machines in mitochondria

**DOI:** 10.1186/s12915-018-0607-3

**Published:** 2018-11-22

**Authors:** Markéta Petrů, Jeremy Wideman, Kristoffer Moore, Felicity Alcock, Tracy Palmer, Pavel Doležal

**Affiliations:** 1 Department of Parasitology, Faculty of Science, BIOCEV, Charles University, Průmyslová 595, 252 50 Vestec, Czech Republic; 2 Wissenschaftskolleg zu Berlin, Wallotstrasse 19, 14193 Berlin, Germany; 3 Department of Biochemistry and Molecular Biology, Dalhousie University, PO Box 15000, Halifax, Nova Scotia B3H 4R2 Canada; 4 Division of Molecular Microbiology, School of Life Sciences, University of Dundee, Dundee, DD1 5EH UK; 5 Department of Biochemistry, University of Oxford, Oxford, UK

**Keywords:** Mitochondrial evolution, TAT translocase, Protein transport, Hydrophobicity

## Abstract

**Background:**

Bacteria and mitochondria contain translocases that function to transport proteins across or insert proteins into their inner and outer membranes. Extant mitochondria retain some bacterial-derived translocases but have lost others. While BamA and YidC were integrated into general mitochondrial protein transport pathways (as Sam50 and Oxa1), the inner membrane TAT translocase, which uniquely transports folded proteins across the membrane, was retained sporadically across the eukaryote tree.

**Results:**

We have identified mitochondrial TAT machinery in diverse eukaryotic lineages and define three different types of eukaryote-encoded TatABC-derived machineries (TatAC, TatBC and TatC-only). Here, we investigate TatAC and TatC-only machineries, which have not been studied previously. We show that mitochondria-encoded TatAC of the jakobid *Andalucia godoyi* represent the minimal functional pathway capable of substituting for the *Escherichia coli* TatABC complex and can transport at least one substrate. However, selected TatC-only machineries, from multiple eukaryotic lineages, were not capable of supporting the translocation of this substrate across the bacterial membrane. Despite the multiple losses of the TatC gene from the mitochondrial genome, the gene was never transferred to the cell nucleus. Although the major constraint preventing nuclear transfer of mitochondrial TatC is likely its high hydrophobicity, we show that in chloroplasts, such transfer of TatC was made possible due to modifications of the first transmembrane domain.

**Conclusions:**

At its origin, mitochondria inherited three inner membrane translocases Sec, TAT and Oxa1 (YidC) from its bacterial ancestor. Our work shows for the first time that mitochondrial TAT has likely retained its unique function of transporting folded proteins at least in those few eukaryotes with TatA and TatC subunits encoded in the mitochondrial genome. However, mitochondria, in contrast to chloroplasts, abandoned the machinery multiple times in evolution. The overall lower hydrophobicity of the Oxa1 protein was likely the main reason why this translocase was nearly universally retained in mitochondrial biogenesis pathways.

**Electronic supplementary material:**

The online version of this article (10.1186/s12915-018-0607-3) contains supplementary material, which is available to authorized users.

## Introduction

Mitochondria evolved from a single alphaproteobacterial ancestor [1]. The transformation of the endosymbiont into an organelle involved the redesign of its membranes and the loss of much of its original genetic information [2, 3]. A key step in this transformation was the evolution of the protein transport machinery that allowed host proteins to be integrated into the evolving organelle. The evolution of these protein import pathways was a complex process, some pathways arose as eukaryotic novelties, whereas some were cobbled together using ancestral bacterial protein transport pathways [4].

Bacteria contain three translocases that function to transport distinct subsets of proteins across or into the inner membrane (IM). In bacteria, SecYEG is the main translocase that post-translationally [5] or co-translationally [6] transports unfolded polypeptides. YidC assists SecYEG in the assembly of membrane proteins or functions as the insertase on its own [7]. The twin-arginine translocase (TAT) allows transport of fully folded or even multi-subunit complexes across the inner membrane, which participate in diverse cellular processes such as respiration, photosynthesis and cell division [8].

In eukaryotes, while the function of the YidC homologue (Oxa1) is conserved, the function of SecY and TAT homologues is less clear. Unlike SecY and TAT, Oxa1 has a highly conserved primary structure and is encoded in the nuclear genome of all eukaryotes retaining a mitochondrial genome. It functions by mediating the co-translational insertion of membrane proteins translated on the mitochondrial ribosome [9]. The evolutionary path and the function of mitochondrial (mt) TAT and Sec still remain largely unknown [10]. So far, mitochondrial SecY has been identified only in the mt genome of jakobids [11–13]. Conversely, although patchily distributed, TAT subunits have been found in the mt genomes of multiple eukaryotes ranging from jakobids to plants and even animals (i.e. sponges) [13–15]. The broad evolutionary distribution of TAT in eukaryotes implies functional significance, yet, protein transport by mitochondrial Tat translocase has not been investigated.

In bacteria, the TAT machinery is built from two types of proteins. Polytopic TatC comprising six transmembrane domains (TMDs) is the largest TAT protein and serves as the main component of the docking complex [16–19]. Different bacterial species contain between one and three paralogous TatA-type proteins, called TatA, TatB and TatE, each of which has an N-terminal TMD and a C-terminal helical domain exposed to the cytoplasm [8, 20].

Protein translocation in proteobacteria requires TatA, TatB and TatC and is initiated by the interaction of cargo signal peptide with the docking complex composed of TatB and TatC. This step is followed by TatC-mediated insertion of the signal peptide into the membrane [21] and subsequent oligomerisation of TatA subunits, which are assumed to form a transient translocation pore around the substrate [22]. In some species, the additional TatE subunit may play an analogous role to TatA, although it is not required for the normal function of the translocase [23]. The minimal functional TAT translocase in some Gram-positive bacteria and Archaea consists only of TatA and TatC subunits [24]. The TatA in these organisms is thought to fulfil the roles of both proteobacterial TatA and TatB proteins.

Eukaryotes are also known to contain TAT components. These proteins are always associated with mitochondria or plastids, the organelles of bacterial ancestry. While the TAT translocase was initially characterised in chloroplast thylakoids and has been studied in further detail [25, 26], only a single study on the mitochondrial TAT translocase has been published so far [14].

Here, we investigate the evolution and function of mitochondrial TAT translocases. By mapping the distribution of mitochondrial TAT subunits in eukaryotes we define three types of TAT-derived machineries with different constituent components (TatAC, TatBC and TatC-only). Using the TatAC machinery of the jakobid *Andalucia godoyi*, we demonstrate partial complementation of *Escherichia coli* TAT. The most common type of eukaryotic TAT is defined by the presence of only mitochondrially encoded TatC. We show that the TatC-only machinery from the heterolobosean *Naegleria gruberi* cannot complement *E. coli* TAT. In vivo, the protein forms stable high molecular weight complexes in the mitochondrial membrane. While TatC has been lost from the mitochondrial genome at least 21 times during the evolution of eukaryotes, we could not find any evidence for mitochondrial TatC in any nuclear genome. We discuss possible constraints of the gene transfer and propose a mechanistic solution, which has occurred for chloroplast TatC in the green plastid lineage.

## Results

### Distribution of mitochondrial TAT in eukaryotes

**Fig. 1:**
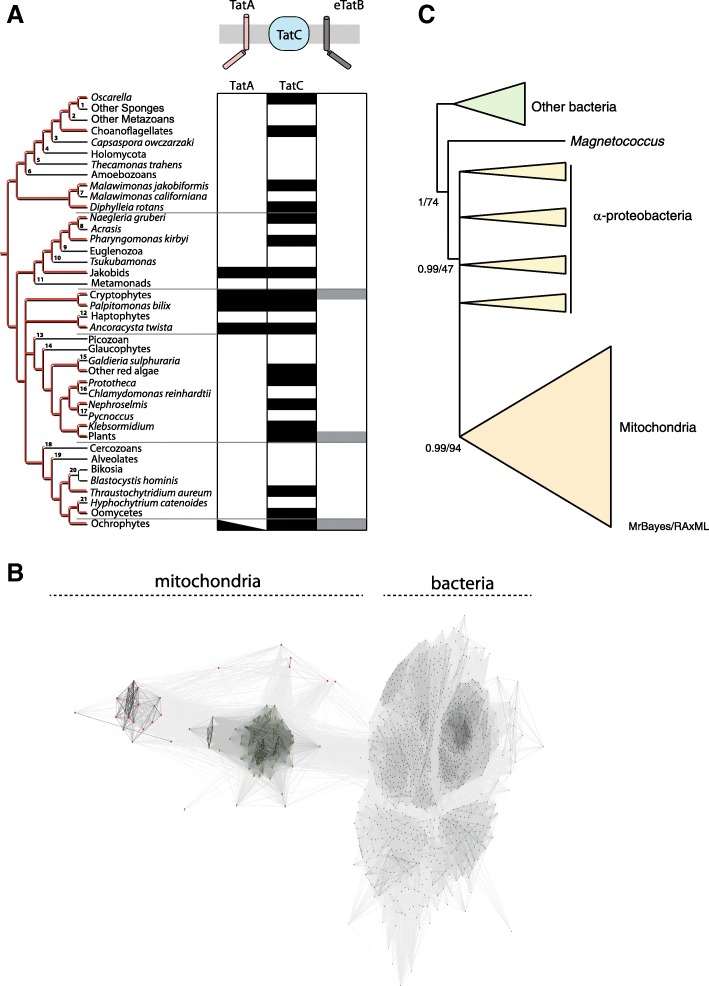
Evolution of the twin arginine translocase (TAT) in eukaryotes. **a** Distribution of mitochondrial Tat components across eukaryotes depicts at least 21 independent losses of the signature TatC component. Previously identified TatA and TatC proteins were collected from sequenced mitochondrial genomes. Their evolutionary distribution was mapped across a consensus tree of eukaryotes. Black rectangles indicate a gene’s presence or absence in a particular mitochondrial genome. The black triangle in ochrophytes indicates that TatA has been lost on numerous occasions in this lineage. The grey rectangle depicts the lineages, where putative nucleus-encoded TatB was found. **b** Representation of CLANS analysis of 1111 TatB protein sequences demonstrating separation of bacterial and mitochondrial TatB sequences (*P* value threshold of 10^−10^). Bacterial sequences are shown as black dots and mitochondrial proteins of plants and ochrophytes as green and red dots, respectively. **c** Schematic tree showing that mitochondrial TatC is monophyletic. Predicted protein sequences of TatC from eukaryotes and a subset of prokaryotes, focusing on alphaproteobacteria, were aligned using MUSCLE and subjected to phylogenetic reconstructions using MrBayes for computing posterior probability and RaxML for maximum likelihood (see the ‘Materials and methods’ section for more detail and Additional file 1: Figure S1 for complete tree). Support values are as inset (MrBayes/RaxML)

In order to gain a comprehensive picture of the distribution of the mitochondrial TAT pathway, available eukaryotic nuclear and mitochondrial genomes representing all major eukaryotic supergroups were searched for the presence of TatA-related and TatC proteins. TAT components could not be found in amoebozoans but could be found in all other major eukaryotic supergroups (opisthokonts, excavates, SAR and archaeplastids), plus cryptophytes and the newly identified protist *Ancoracysta twista* (Fig. 1a). In the vast majority of TAT-containing eukaryotic species, the presence of TAT machinery is limited to only TatC. TatA is only present in lineages that contain TatC, including jakobids [27], cryptists (including *Palpitomonas bilix*) [28], ochrophytes [29] and *A. twista* [30] (Fig. 1a).

In all cases, the mitochondrial TatA and TatC components are encoded by the mitochondrial genome. However, the gene for a putative plant mitochondrial TatB was recently identified on the plant nuclear genome [14]. The product of this nucleus-encoded gene is localised to mitochondria and has been suggested to be part of a stable high molecular weight TatBC complex. The gene is slightly longer than its prokaryotic counterparts, and the role of the whole mitochondrial complex remains unknown. We searched the eukaryotic genomes for homologues of this mitochondrial TatB and identified several homologues in stramenopiles (like oomycetes) and cryptists (Fig. 1a), where it was also encoded in the nucleus. The eukaryotic and prokaryotic TatB proteins share a longer amphipathic α-helix exposed to the cytoplasm/mitochondrial matrix when compared to TatA proteins [31] and also conserved signature residues, including the essential glutamate residue in the TMD (Additional file 1: Figure S1). All of the TatB-positive eukaryotes carry mitochondrial encoded TatC, which provides additional support for their possible function in mitochondria. However, we could not identify the common presence of all three mitochondrial TatABC subunits (Additional file 2: Table S1).

A phylogenetic reconstruction of TatB proteins, which could resolve the origin of the eukaryotic proteins in different lineages of eukaryotes, was hampered by their very small size. Instead, a CLANS analysis, which is based on mutual blast comparisons, demonstrated that bacterial and (nucleus-encoded) mitochondrial TatB form distinct groups of sequences (Fig. 1b), suggesting that, although structurally related, no obvious horizontal gene transfer has occurred between these proteins. The analogous clustering analysis of bacterial and mitochondrial TatAs showed great divergence of the mitochondria-encoded proteins as they remained scattered without clear mutual affinity (Additional file 1: Figure S2). While parsimony would suggest that mitochondrial TatBs come from a common ancestral bacterial TAT, the reason why only TatBs and never TatAs are found in the nuclear genome remains a mystery.

The patchy distribution of mitochondrial TatC in eukaryotes indicates that the gene has been lost multiple times during evolution. To ensure that the patchy distribution is not seen due to repeat acquisition by lateral gene transfer (LGT) from bacteria, we reconstructed the phylogeny of TatC proteins from mitochondria and bacteria. A monophyletic mitochondrial TatC was recovered with high full support by both Bayesian (MrBayes) and maximum likelihood (RaxML) methods suggesting that mitochondrial TatC was derived from a single alphaproteobacterial origin, ruling out multiple LGTs from bacteria (Fig. 1c, Additional file 1: Figure S3). Mapping the presence and absence of TatC across a consensus tree of eukaryotes revealed that at least 21 losses occurred over the course of eukaryote evolution (Fig. 1a).

### Three arrangements of TAT-derived mitochondrial machinery

The presence of TatABC components in almost all alphaproteobacteria [32] and the proposed origin of mitochondria within this group [33] indicate that the early mitochondrion also possessed a three-component translocase. Thus, since the TAT machinery is endosymbiont-derived, regardless of the distribution of TAT machinery, the last eukaryotic common ancestor (LECA) must have also contained a complete three-component translocase. We can conclude that the absence of any component must be due to loss.

Our comparative analysis suggests that evolution has generated different TAT-derived arrangements, which can be distinguished in extant mitochondria: (I) mitochondria-encoded TatA and TatC subunits reminiscent of the minimalist bacterial TAT translocase [24], (II) mitochondria-encoded TatC accompanied by nuclear-encoded TatB protein [14] and (III) mitochondria-encoded TatC-only systems. In regard to the non-uniform occurrence of mitochondrial Tat components, it seems very possible that a complete set of all three components will be identified when more nuclear genome sequences become available. Consequently, two independent questions have been raised: (1) has mitochondrial TAT retained its ancestral function in any of its derived forms? and (2) what is the current function of TAT in extant mitochondria?

### *Andalucia godoyi* TatAC complex retains ancestral functionality but TatC-only complex of *N. gruberi* and *Malawimonas jakobiformis* does not

TatBC complexes have been recently investigated in plants, whereas the other two TatAC and TatC-only complexes have not yet been investigated [14]. Therefore, we sought to characterise the TatAC complex of *Andalucia godoyi* and the TatC-only complexes of *Naegleria gruberi* and *Malawimonas jakobiformis*.

**Fig. 2:**
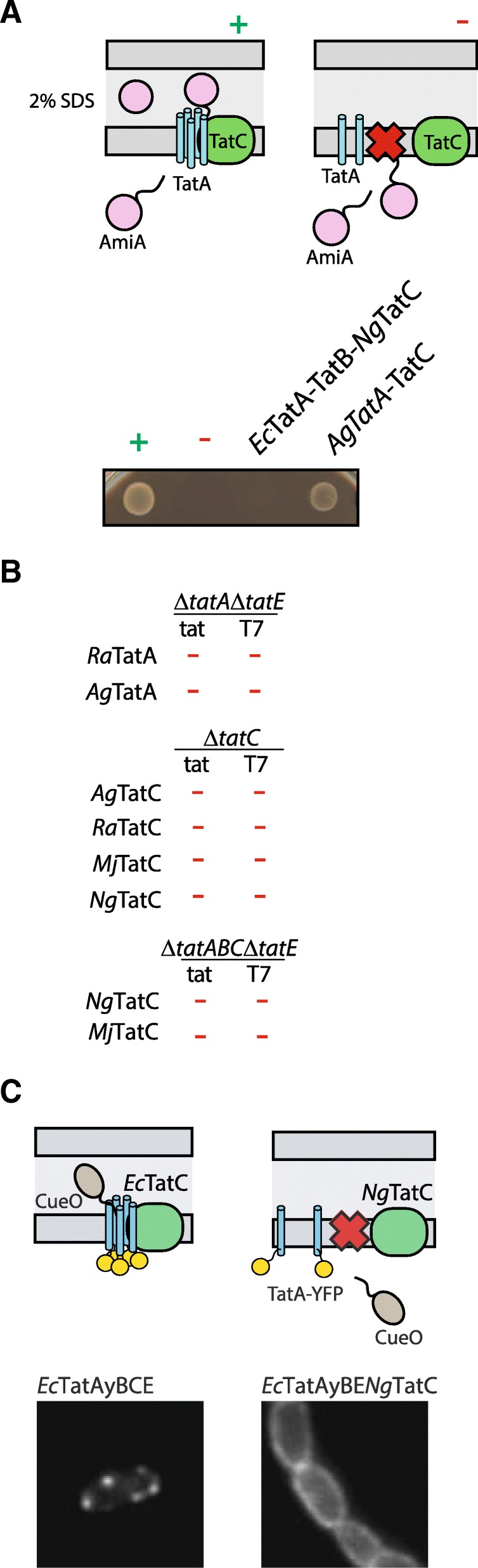
Mitochondrial Tat proteins of *A. godoyi* but substitute for the function of *E. coli* TAT pathway. **a** Diagrams depict the principle of the complementation assay aimed at the translocation of TAT substrate (AmiA) across the inner *E. coli* membrane. + (pTAT101 plasmid carrying complete *tat* operon) and − (pTAT101 without *tat* operon) represent the positive and negative controls, respectively. In all strains (MCDSSAC Δtat), mature domain of AmiA with SufI Tat signal peptide was expressed. While *A. godoyi* mitochondrial TatA and TatC (expressed from pTAT101) could functionally replace *E. coli* proteins, *N. gruberi* TatC could not rescue the function when expressed in a synthetic operon with *E. coli* proteins. **b** Individual TatA and TatC were expressed from plasmids containing promotor of varying strength (tat or T7 promotor) in corresponding *E. coli* mutant (top line of each panel). None of the gene could functionally replace the bacterial protein or, in case of TatC, the whole TAT system. *Ag—A. godoyi*, *Ng—N. gruberi*, *Ra—R. americana*, *Mj—M. jakobiformis.* **c** Diagrams depict the clustering of YFP-tagged TatA (TatAy) induced upon the overexpression TAT substrate (CueO). Replacing *E. coli* TatC with *Ng*TatC abolished the formation of the active translocase clusters

*A. godoyi* is a deep-branching jakobid [34] with the greatest mitochondrial coding capacity known to date [13]. All jakobids are experimentally very challenging organisms, without any established biochemical, cell biological or molecular procedures. In order to test the function of *A. godoyi* and *N. gruberi* TAT machinery, we used a heterologous expression system in *E. coli* specifically engineered to characterise the function of the TAT translocase [35, 36]. Briefly, the *E. coli* strains lacking all or a subset of *tat* gene(s) were transformed with plasmids carrying individual *tatA* or *tatC* genes or a synthetic operon containing both *A. godoyi* genes. The functionality of the translocase was monitored by TAT-mediated translocation of AmiA, a cell wall hydrolase, the function of which is essential for the growth of the transformants on SDS-containing media [37]. Introduction of a synthetic operon of *A. godoyi tat* genes resulted in transport of AmiA carrying the signal peptide of SufI, another *E. coli* TAT substrate, as manifested by the restored growth of the strain on selective media (Fig. 2a). However, neither individual TatA nor TatC could complement the growth defect of the corresponding *E. coli* mutant strain when produced under natural (TAT) or strong (T7) promoters (Fig. 2b). These data strongly suggest that mitochondrial TAT proteins of *A. godoyi* represent functional machinery capable of substituting the role of the *E. coli* translocase.

The combination of mitochondrial and bacterial proteins did not result in a functional complex, likely due to the incompatible protein-protein interfaces. Negative results were also obtained using individual components of another jakobid *Reclinomonas americana* (Fig. 2b), further suggesting that mitochondrial and bacterial machineries are not compatible to combine into chimeric translocases.

Similarly, *Ng*TatC, representing the TatC-only system, was introduced into specific *E. coli* mutant strains lacking *tatC* gene or the whole *tat* operon. In these strains, the gene was expressed either alone or as a part of synthetic operon containing *E. coli tatAB* genes. The premise for these combinations was that TatC-only system may carry out the role of full TAT translocase in mitochondria.

However, *Ng*TatC was able to restore neither the function of TatC nor the complete TAT system in bacteria (Fig. 2b). To test whether another TatC-only system might restore the function of the *E. coli* mutant, analogous experiments were performed with TatC from *Malawimonas jakobiformis*. Similar to *N. gruberi*, *M. jakobiformis* also encodes only TatC (*Mj*TatC) in the mt genome, but according to recent phylogenetic analyses [38, 39], it belongs to a different deep-branching clade of eukaryotes. Like the TatC from *N. gruberi*, *Mj*TatC also failed to substitute for the *E. coli* protein in the AmiA translocation assay.

However, the expression of *Mj*TatC in *E. coli* was not confirmed due to the lack of a protein tag or a specific antibody, as it was done for *Ng*TatC (see below). Given that both genes were similarly codon-optimised for the expression in *E. coli,* we assumed that, in both cases, the lack of complementation is caused by the inability of TatC to recognise a bacterial TAT substrate and trigger the translocation in concert with *E. coli* TatB and TatA proteins. Indeed, when *Ng*TatC was expressed in place of the *E. coli* protein in bacteria carrying YFP-tagged TatA, no in vivo formation of transient TatA complexes was observed in contrast to the control cells, in which TatA complexes could be observed as fluorescent clusters (Fig. 2c).

### *Naegleria gruberi* TatC localises to mitochondria-enriched fraction in a high molecular weight complex

**Fig. 3:**
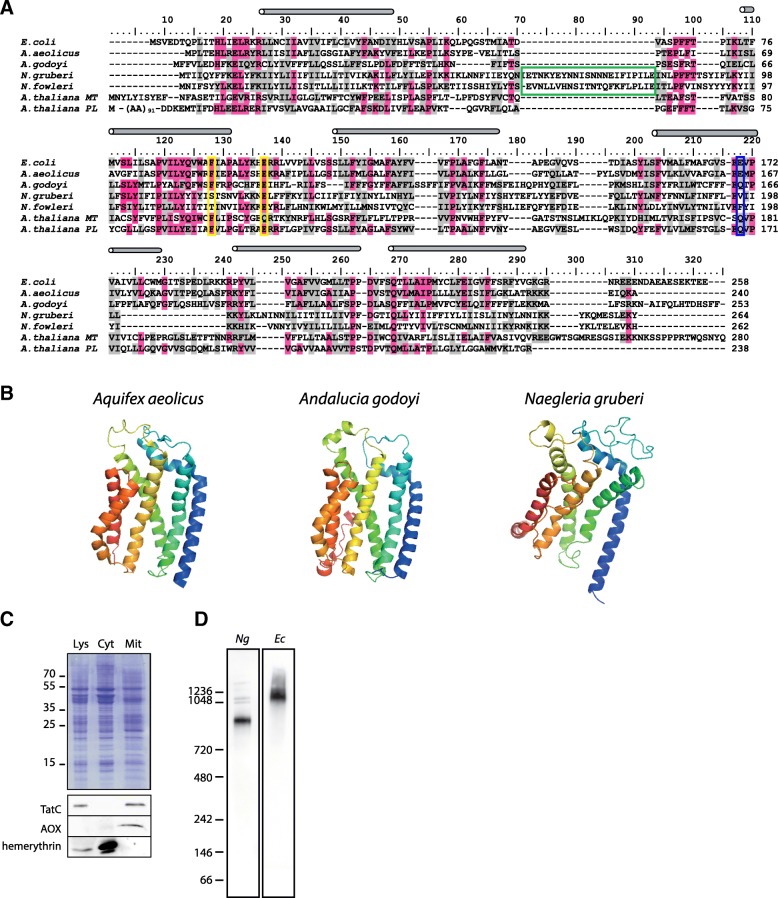
Mitochondrial TatC of *Naegleria gruberi*. **a** Protein sequence alignment of bacterial and mitochondrial TatC proteins. The blue rectangle highlights the conserved glutamate/glutamine residue in the center of TatC cavity. The yellow rectangle depicts the position of conserved phenylalanine and glutamate residue of the signal peptide binding site. The green rectangle highlights the insertion common to heterolobosean TatC, and grey cylinders depict the transmembrane domains. Identical and similar residues are highlighted in pink and grey colour, respectively. The threshold for shading was 50%. The C-terminal peptide used for antibody generation is underlined. **b** The comparison of structure of bacterial TatC protein from *A. aeolicus* [17] and TatC from *A. godoyi* and *N. gruberi* obtained by I-TASSER [69]. *N. gruberi* protein shows overall relaxed structure with unclear arrangement of the fifth membrane spanning domain **c** Western blot analyses of *N. gruberi* cellular fractions. **d** *Ng*TatC forms high molecular weight complexes on BN-PAGE in mitochondria (*Ng*) and also when expressed with C-terminal HA-tag in *E. coli* (*Ec*)*.* In both cases, the membranes were solubilised in 2% digitonin

*Ng*TatC is rather divergent compared to other mitochondrial (22% identity to *A. godoyi*) and bacterial TatC proteins (14% and 19% identity to *E. coli* and *Aquifex aeolicus*, respectively). This divergence could account for its inability to complement *E. coli* TAT. For instance, *Ng*TatC and other heterolobosean proteins lack the key functional glutamate/glutamine residue (E170; *E. coli* numbering) of the central cavity, and the conserved phenylalanine residue (F94) involved in signal peptide binding was replaced by polar amino acid (Fig. 3a). In addition, a heterolobosean-specific insertion (of 22–23 AA) between the first and second transmembrane domains was detected (Fig. 3a). The overall divergence of the sequence is likely responsible for the relaxed predicted structure of *Ng*TatC, when compared to the TatC of *A. aeolicus* or the modelled structure of the *A. godoyi* protein (Fig. 3b).

In order to test whether *Ng*TatC is expressed and localised in mitochondria, a polyclonal antibody was raised against the very C-terminus of the protein, which is predicted to be exposed to the mitochondrial matrix. Immunolabelling of the *N. gruberi* cellular fractions specifically detected a band of 35 kDa in the cell lysate and mitochondria-enriched fraction, but not the cytosolic fraction. Hemerythrin- and alternative oxidase (AOX)-specific antibodies [40] were used to detect cytosolic and mitochondrial markers, respectively. Thus, we conclude that our antibody is detecting TatC in mitochondria (Fig. 3c).

The mitochondria-enriched fraction was further solubilised in digitonin and resolved on blue native (BN) PAGE. The western blot showed TatC to be present in a high molecular weight complex of approximately 900 kDa (Fig. 3d), which was highly resistant to the increasing detergent concentration (0.5–2%) and did not disassemble into smaller protein species. The mitochondrial TatC-containing complex in *N. gruberi* is larger than the bacterial or plastidial TatBC receptor complexes, which appear as a 440- or 700-kDa complex on BN PAGE [18, 41], respectively. Within the bacterial and plastidial TatBC receptor complexes, TatB and TatC are of yet unspecified stoichiometry [42, 43], but both systems require the oligomerisation of TatC for proper function [44]. That *Ng*TatC is prone to oligomerise was also demonstrated by the expression of the hemagglutinin (HA)-tagged protein in *E. coli*, where even a larger complex was detected on BN-PAGE (Fig. 3d). Similarly, the plant mitochondrial TatBC-containing complex was also shown to migrate as a very large complex of 1500 kDa [14], suggesting that TAT translocases in bacteria and plastids as well as mitochondrial TAT-derived machineries may be of different architecture.

### Search for the mitochondrial TAT substrate

The activity of mitochondrial TAT in *A. godoyi* prompted us to search for putative TAT substrates. With regard to the presumed topological conservation of the translocase in bacterial and mitochondrial membranes, a putative substrate of the mitochondrial TAT translocase would be recognised by the receptor complex in the mitochondrial matrix and translocated into the intermembrane space. Such substrate(s) could originate from two distinct groups of proteins: either from mitochondria-encoded proteins, which would present the twin arginine signal peptide after their translation on the mitoribosome, or from nucleus-encoded proteins targeted to the mitochondrial matrix, which would expose the signal peptide upon the removal of their mitochondria targeting sequence. We analysed all 72 proteins encoded by the mt genome of *A. godoyi* using the TatP prediction algorithm [45], but no clear candidate proteins could be detected. This finding was further supported by the comparisons of mitochondrial genomes carrying/lacking Tat components [33] with the assumption that putative substrate of the TAT pathway would co-occur with subunits of the translocase. The fact that no such gene could be identified indicates that the substrate of mitochondrial TAT may be among the nucleus-encoded mitochondrial proteins. The bioinformatic search for such proteins is currently hindered by the lack of complete annotated *A. godoyi* genome.

Recently, the Rieske protein has been suggested as a putative mitochondrial TAT substrate [15]. This iron-sulfur cluster-containing protein was identified as a substrate of the plastid and bacterial TAT translocase [46–48]. Through analysis of a set of Rieske proteins, which showed a direct positive correlation between the presence of a Tat component in the mitochondrial genome and a twin arginine motif in the Rieske proteins, it was hypothesised that the analogous function may be performed by the mitochondrial TAT [15]. However, in yeast and possibly in other opisthokonts, Bcs1 AAA-ATPase was shown to mediate the translocation of the Rieske protein [49].

To test the possible role of TAT in Rieske protein translocation, we made a synthetic construct carrying the N-terminal part of *A. godoyi* mitochondrial Rieske corresponding to its bacterial counterparts fused to mature AmiA. The premise was that *A. godoyi* TAT machinery may recognise the signal peptide of its natural substrate even in the heterologous system, which could be monitored by the translocation of AmiA during the growth assay.

**Fig. 4:**
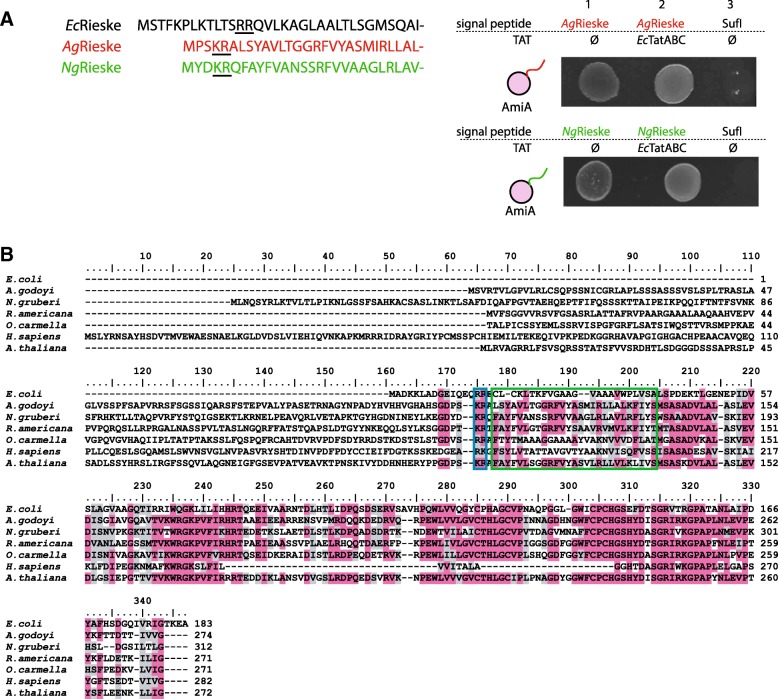
Rieske proteins as putative substrate of mitochondrial TAT. **a** The experimental signal peptides of *A. godoyi* and *N. gruberi* Rieske proteins. *Ag*/*Ng*Rieske signal sequences fused to mature AmiA expressed from pSU18 are translocated through bacterial membrane in both absence (1; pTH19kr) and presence (2; pTAT101) of TAT in MCDSSAC Δtat strain. (3) SufI signal sequence fused to mature AmiA expressed from pSU18 is not translocated in the absence of TAT (pTH19kr) in MCDSSAC Δtat strain. **b** Protein sequence alignment of the Rieske protein orthologues shows the presence of putative twin arginine motif in both TAT-positive (TatAC in *A. godoyi* and *R. americana,* TatBC in *A. thaliana*, TatC-only in *N. gruberi* and *O. carmella*) and TAT-negative eukaryotes (*H. sapiens*). The blue rectangle highlights the presence of twin arginine (RR) or derived (KR and RK) motifs, and the green rectangle depicts the transmembrane domain. Identical and similar residues are highlighted in pink and grey colour, respectively. The threshold for shading was 50%

While the presence of the chimeric protein supported the growth of bacteria, which indicated successful AmiA translocation, we found that the translocation was not dependent on the presence of TAT (Fig. 4a). The same result was obtained when a chimeric construct based on the *N. gruberi* Rieske protein was co-produced in the presence of synthetic operon containing *Ng*TatC (Fig. 4a).

We assumed that the designed hydrophobic synthetic signal peptide was recognised and translocated as Sec substrate instead of TAT pathway, as recently observed for mutant *E. coli* Tat signal peptide [50]. Such crosstalk between Sec and Tat pathways observed in bacteria did not allow us to test the actual interaction between mitochondrial Rieske and TAT pathway in bacteria. However, this situation cannot occur in the vast majority of mitochondria, as both mitochondrial Sec and Tat translocases have been found only in several jakobid species except *A. godoyi* [11, 13].

Comparison of the mitochondrial Rieske proteins confirmed the presence of twin arginine motif or its derived versions (RK, KR) at the conserved site [15]. It was demonstrated earlier that these imperfect motifs can be also transported via bacterial TAT [51]. However, the presence of these motifs does not correlate with the presence or the absence of Tat components in the mitochondria (Fig. 4b). It is thus possible that the same motif, which is used by the TAT pathway in bacteria may serve other purposes in mitochondria. The positively charged residues may perhaps rather define the positioning of the adjacent transmembrane anchor (Fig. 4b) of the Rieske protein in the inner mitochondrial membrane, instead of serving as a signal for TAT-dependent translocation.

To conclude, our experiments show for the first time that a mitochondrial-encoded TatAC complex constitutes a functional translocase. Nonetheless, the nature of the cargo transported out of mitochondrial matrix and perhaps into other cellular compartments of the eukaryotic cell remains unknown. Hence, future genomic and experimental analyses are necessary to understand the integration of different TAT-derived machineries into the mitochondrial protein transport pathways.

### The preservation or gradual loss of the inner membrane translocases of bacterial origin in eukaryotes

As mentioned above, mitochondrial *tatC* and *tatA* genes could only be identified in the mitochondrial genome and not in the cell nucleus. This means the loss of *tatA* and *tatC* genes from the mt genome was never accompanied by their transfer to the nuclear genome. Several different hypotheses explain the preservation of particular genes in mitochondrial genomes [33, 52, 53]. Among them, high hydrophobicity has been considered as one of the key pressures against the transfer of mitochondrially encoded genes to the nucleus.

**Fig. 5:**
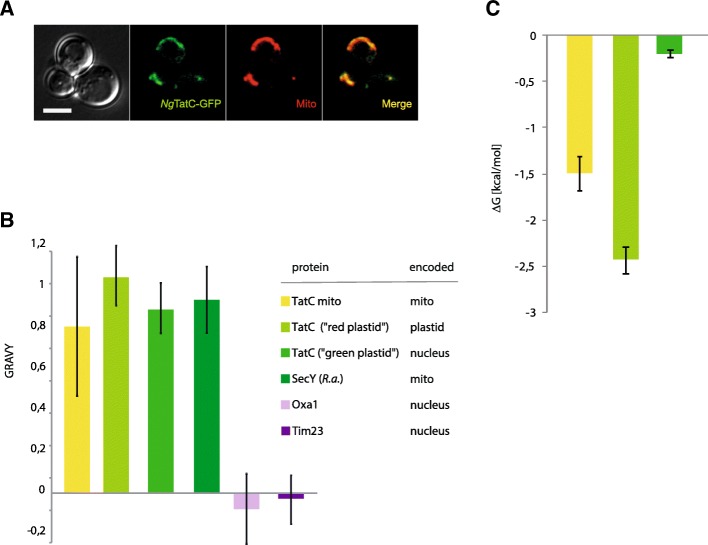
Physical properties of mitochondria-encoded protein translocases. **a** Allotopic expression of *Ng*TatC with the C-terminal GFP in *S. cerevisiae*. **b** The plot of GRAVY values of mitochondrial and plastid TatC proteins. **c** The plot of free membrane insertion energy (Δ*G*) of mitochondrial and plastid TatC proteins. The error bars represent standard deviation of the calculated values

The experimental allotopic expression of mitochondria-encoded proteins often results in protein degradation or mistargeting to other compartments [54, 55]. Surprisingly, when codon-optimised *ngtatC* with a C-terminal GFP was expressed in *Saccharomyces cerevisiae*, the protein localised to mitochondria in some yeast cells even without an N-terminal targeting sequence (Fig. 5a). However, the vast majority of cells (≈ 95%) were devoid of any fluorescent signal, which likely indicated difficulty in handling such a hydrophobic protein. Similarly, *Ng*TatC without any tag was expressed in very low amounts in *S. cerevisiae* mitochondria (Additional file 1: Figure S4), making further characterisation of the protein (i.e. targeting, folding and topology) impossible.

In the context of mitochondrial gene transfer, the term ‘hydrophobicity’ can describe two different physical characteristics [55]: first, the overall hydrophobicity of the polypeptide, which can cause cytoplasmic aggregation if not bound by molecular chaperones [56], and second, the hydrophobicity of individual TMDs in the nascent polypeptide, which can be recognised by the SRP pathway [57] and incorrectly directed to the endoplasmic reticulum (ER) membrane.

In order to assess the actual hydrophobicity of mitochondrial TatC proteins, the grand average of hydropathy (GRAVY) was calculated. Mitochondrial TatC proteins were identified as highly hydrophobic proteins, greatly above the GRAVY values of typical polytopic inner mitochondrial membrane proteins like Tim23 and Oxa1 (Fig. 5b). Proteins as hydrophobic as mitochondrial TatCs would be expected to aggregate in the cytoplasm unless greatly assisted by molecular chaperones.

The propensity of a protein to be recognised by the SRP pathway in the cytoplasm and subsequently inserted into the ER membrane can be estimated by the procedure described by Björkholm et al. [58]. It takes into account free (membrane) insertion energy (Δ*G*, kcal/mol) of the first TMD appearing from the ribosome [59] and the presence of the following C-terminal part of the protein longer than 120 amino acid residues.

Plotting the calculated Δ*G* values of mitochondrial TatC TMDs suggests a strong tendency to be inserted into a membrane (− Δ*G*), which, together with a polypeptide length over 120 residues, strongly suggests that they would be preferentially recognised by SRP and mistargeting to the ER (Fig. 5c). Similarly, the known ancestral mitochondrial orthologues of the main bacterial SecY translocase, which were identified in the mitochondrial genome of jakobids, are highly hydrophobic protein, which would also be difficult to re-translocate into the organelle [11, 12].

## Discussion

The adaptation of the endosymbiotic bacterium into the genetically dependent mitochondrion relied on reversing the protein flow across the symbiont’s inner and outer membranes. Multiple bacterial protein secretion pathways were replaced by mitochondrial protein import machines; however, a few have been retained by the organelle [4]. In addition to the orthologous core components of BAM and SAM complexes, which assemble the β-barrel proteins in the outer membrane of bacteria and mitochondria, respectively, orthologues of all three bacterial inner membrane translocases YidC/Oxa1, TAT and Sec can be found in mitochondria [13].

In comparison to TAT and Sec, both with highly limited distribution across eukaryotes, only Oxa1 has been maintained in the essential mitochondrial protein transport pathways [60]. However, given that diverse eukaryotes have retained functional TAT complexes in their mitochondria for over a billion years of evolution, evolutionary theory dictates that these translocases must be functionally important. Nonetheless, it is still unclear exactly what these functions are.

While SecY and TatC are always encoded by the mitochondrial genome, Oxa1 is always encoded in the nucleus and posttranslationally translocated into mitochondria [60]. This means that while Oxa1 was able to be transferred to the nucleus and then retargeted to the mitochondria, SecY and TatC were never able to make that transition. Our comparison of Oxa1 to TatC and SecY suggests that it was the much lower hydrophobicity of the Oxa1 which enabled its nuclear transfer (Fig. 5b).

Interestingly, although mitochondrial TatC has never been transferred to the nucleus, chloroplast TatC has been successfully transferred in some lineages. Analyses of plastid TatC proteins showed that the proteins from both red and green plastids remained highly hydrophobic and in the case of red plastid TatC even exceeds the GRAVY values of mitochondrial proteins (Fig. 5b). However, according to free insertion energy (Δ*G*) calculations (Fig. 5c), the structure of the first TMD of green plastid TatC is much more favorable to escape SRP recognition, thus allowing the transport of TatC to the plastid.

The transfer of mitochondrial genes to the cell nucleus resulted in the formation of the eukaryote-specific protein import complexes in the inner mitochondrial membrane built around proteins from the Tim17 family [61]. That multiple eukaryotic lineages retained TatC in their mitochondrial genome without the pore-forming TatA subunit indicates a yet unknown mitochondria-specific adaptation of TAT and illustrates the gradual loss of its importance in mitochondrial biology. On the other hand, with the exception of the Rieske protein, there is currently no record of the export of proteins by any means across the inner mitochondrial membrane, which may indicate some yet unknown fundamental functional constrains linked to the process. In this context, Oxa1 in contrast to TAT and Sec translocases forms only an incomplete hydrophilic cavity and does not form a continuous channel across the membrane [62]. It is thus likely that the unique function of TAT, transporting even fully folded holoproteins, may thus not be compatible with the function of current mitochondria. For our future understanding of the actual function of mitochondrial TAT-derived machineries, the establishment of tractable genetic system in some of the diverse protist lineages is necessary.

## Conclusions

The evolution of protein transport pathways in mitochondria has involved independent gains and losses of components of the inner and outer protein translocases. Here, we show that the TAT translocase of alphaproteobacterial ancestry has undergone a complex evolutionary history in eukaryotes. We demonstrate that the jakobid *A. godoyi* retains a functional TatAC complex capable of rescuing TAT-deficient *E. coli*, unlike the TatC-only complexes of *N. gruberi* and *M. jakobiformis*. Unfortunately, eukaryotic substrates stay elusive, and therefore, the function of all mitochondrial TAT complexes remains unknown. We suggest that the hydrophobicity of TatC made its transfer to the nuclear genome impossible. But why the complex is dispensable in so many eukaryotic lineages remains to be determined.

## Materials and methods

### Bioinformatic analysis

Homologues of TatA and TatC were identified in a subset of available mitochondrial genomes using a combination of BLASTp, tBLASTn [63], locally installed [64], and the online version of HMMer [65] and mfannot annotations (http://megasun.bch.umontreal.ca/cgi-bin/mfannot/mfannotInterface.pl.) Initially, local HMMer search against 60 selected predicted eukaryotic proteomes (Additional file 2: Table S2), representing the major eukaryotic supergroups, was performed followed by the online search against UniProtKB. The Pfam seed alignments were used as a query.

In case of putative mitochondrial TatB proteins, HMMer search [65] (https://www.ebi.ac.uk/Tools/hmmer/) against UniProtKB was done using plant mitochondrial TatB as a query [14].

To reconstruct the phylogeny of tatC, protein sequences encoded in bacterial and mitochondrial genomes (165 sequences) were aligned using MUSCLE [66] and manually trimmed using Mesquite v2.75 resulting in 220 sites. Maximum likelihood bootstrap values (100 pseudoreplicates) were obtained using RAxML v8.2.10 [67] under the LG model [68] and MrBayes v.3.2.6 [69] for computation of posterior probabilities. MrBayes analyses were run with the following parameters: prset aamodelpr = fixed (LG), mcmcngen = 2,000,000, samplefreq = 1000, nchains = 4, startingtree = random and sumt burnin = 250. Split frequencies and PRSF values were checked to ensure convergence (average SD of split frequencies = 0.035790). The homology model of the three-dimensional structure of *Ng*TatC and *Ag*TatC was built with i-TASSER [70]. The GRAVY values were calculated by http://www.gravy-calculator.de/ and free (membrane) insertion energy (Δ*G*) by http://dgpred.cbr.su.se/ [59]. The transmembrane domains were predicted TMHMM [71]. Eukaryotic TatB secondary structures were modelled by MINNOU (http://minnou.cchmc.org/).

### Construct preparation

*Ag*TatA (YP_007890508.1), *Ag*TatC (YP_007890501.1), *Mj*TatC (NP_066348.1), *Ng*TatC (NP_066540.1), *Ra*TatA (NP_044807.1) and *Ra*TatC (NP_044778.1) sequences were codon optimised for the expression in *E. coli* and synthesised (Thermo Fisher Scientific). For the complementation assays, *tatA* and *tatC* genes with the C-terminal His- or HA-tag on were cloned in pUNIPROM (tat promotor) within 5′ BamHI and 3′ XbaI cloning sites and pBluescript II KS+ (T7 promotor) within 5′ ApaI and 3′ SacI cloning sites (see Additional file 2: Table S3 for details on primers and restriction sites used). The synthetic TAT operon containing *ngtatC* (*ectatA-tatB-ngtatC*) or *agtatAtatC* was synthesised and then cloned into pTAT101 within 5′ BamHI and 3′ PstI cloning sites. For TatA-YFP assay, *ectatB* and *ngtatC* were amplified from *ectatA-tatB-ngtatC* synthetic operon and cloned into p101C*TatBC plasmid in place of *ectatBC* within 5′ BamHI and 3′ PstI cloning sites.

To test *A. godoyi* Rieske protein as a putative substrate of TAT, *Ag*Rieske coding sequence was recovered from the ongoing genome project (Marek Eliáš, unpublished). The sequence was codon optimised for the expression in *E. coli* and commercially synthesised (*agrieskeopt*).

For the generation of chimeric Rieske-AmiA constructs, putative mitochondrial targeting sequences of *Ag*Rieske (residues 2–79) and *Ng*Rieske (2–123) were removed and putative TAT signal sequences (residues 108–134 and 147–172, respectively) were added to mature *E. coli* AmiA. In both constructs, the cleavage site for bacterial signal peptidase was retained. The constructs were cloned into pSU18 within 5′ EcoRI and 3′ XbaI cloning sites.

For the expression of *ngtatCopt* with the C-terminal GFP in *S. cerevisiae*, the gene was cloned into pUG35 within 5′ XbaI and 3′ BamHI cloning sites. For localisation of *Ng*TatC in yeast mitochondria, *ngtatCopt* was cloned into inducible vector pYES2.0 within 5′ BamHI and 3′ XhoI cloning sites.

### *N. gruberi* cultivation and cell fractionation

*N. gruberi* str. NEG-M was axenically cultured in M7 medium with penicillin (10 U/ml) and streptomycin (10 μg/ml) at 27 °C in vented tissue culture flasks. For cell fractionation, one 300-cm^2^ cultivation flask with fully grown *N. gruberi* culture was harvested. Cells were collected by spinning at 1200×*g*/10 min/4 °C. Pellet was washed once in cold 1× PBS and spun down. Cells were resuspended in 2 ml of SM buffer (250 mM sucrose, 20 mM MOPS pH 7.4) supplemented with Roche cOmplete™-EDTA-free Protease Inhibitor Cocktail and sonicated 3 × 15 s (1 s/1 s), 30%. Resulting cell homogenate was then centrifuged at 1500×*g*/10 min/4 °C to remove unbroken cells and at 21,000×*g* to obtain high-speed pellet corresponding to the mitochondria-enriched fraction. The resulting supernatant corresponded to the cytosolic fraction.

The expression of *Ng*TatC was verified by SDS-PAGE and western blot analysis using affinity-purified *Ng*TatC antibody raised against the C-terminal peptide (NIKKYKQMESLEKYC, unique to *Ng*TatC) obtained from Genscript. For Blue Native PAGE (BN-PAGE) analyses, the mitochondria-enriched fraction was solubilised in 0.5, 1 and 2% digitonin, and samples were resolved on 3–12% gel.

### *E. coli* cultivation and complementation assays

*Escherichia coli* was cultivated aerobically in lysogeny broth (LB) or on LB agar. When required, antibiotics were used at the following final concentrations: ampicillin (amp) at 100 μg/ml, kanamycin (kan) at 50 μg/ml, chloramphenicol (cml) at 50 μg/ml and apramycin (apra) at 50 μg/ml. For complementation assays, LB agar with 2% SDS and particular antibiotic was used. Complementation assays were carried out as previously described [37, 72]. See Additional file 2: Tables S4 and S5 for details of *E. coli* plasmids and strains used in the study, respectively.

### Expression of TatC in *S. cerevisiae* and mitochondria isolation

*Saccharomyces cerevisiae* str. YPH499 and INVSc1 was grown on YPD plates at 30 °C and after lithium-acetate transformation [73] on selective medium without uracil (SD-URA) at 30 °C. For the fluorescence microscopy, *S. cerevisiae* str. YPH499 cells transformed with pUG35 carrying *ngtatC* were incubated with MitoTracker Red CMXRos (1:10,000) for 10 min, washed once in PBS and mounted in 2% low-melting agarose. Cells were viewed using an Olympus IX81 microscope and a Hamamatsu Orca-AG digital camera using the cell^R imaging program at × 100 magnification. For mitochondria isolation, *S. cerevisiae* str. INVSc1 transformed with pYES2.0 or pYES2.0 carrying *ngtatC* was harvested after induction of *ngtatCopt* expression on galactose selective medium without uracil [SC-URA(gal)]. The mitochondria were isolated as published previously [74]. Briefly, 250 ml of the cells (OD_600_ = 1) was collected by spinning at 5000×*g*/5 min/4 °C. Pellet was washed once in 1× PBS and spun down. Cells were resuspended in TRIS-DTT buffer (0.1 M Tris-SO4 pH 9.4; 10 mM DTT) and incubated 15 min/30 °C and, after, spun down at 5000×*g*/5 min/RT. Pellet was washed once in pre-warmed 1.2 M sorbitol buffer (1.2 M sorbitol; 20 mM KPi pH 7.4) and spun down at 5000×*g*/5 min/RT. 2.5 mg zymolyase per gram of cells dissolved in pre-warmed 1.2 M sorbitol buffer was added, and cells were incubated 30 min/30 °C and then spun down at 5000×*g*/5 min/4 °C. From here onwards, everything was kept at 4 °C. Protoplasts were twice resuspended in cold 1.2 M sorbitol buffer and spun down at 5000×*g*/5 min/4 °C. Pellet was resuspended in 7 ml BB6.0 buffer (0.6 M sorbitol; 20 mM K + MES pH 6.0) supplemented with Roche cOmplete™-EDTA-free Protease Inhibitor Cocktail and homogenised using a dounce. Homogenate was spun down at 5000×*g*/5 min/4 °C to get clean supernatant. Finally, the supernatant was spun at 13,000×*g*/20 min/4 °C to obtain high-speed pellet containing mitochondria.

## Additional files

Additional file 1: **Figure S1.** Protein sequence alignment of eukaryotic TatB. **Figure S2.** CLANS analysis of TatA from bacteria and mitochondria. **Figure S3.** Phylogenetic reconstruction of bacterial and mitochondrial TatC. **Figure S4.** Expression of *Ng*TatC in *S. cerevisiae*. (DOCX 1194 kb)

Additional file 2: **Table S1.** eTatB-positive species and the presence of mitochondria-encoded TatA and TatC. **Table S2.** Eukaryotic nuclear genomes used for the initial search of Tat components. **Table S3.** Primers used in the study. **Table S4.** Plasmids used in the study. **Table S5.** Bacterial strains used in the study. (XLSX 21 kb)
