# Supplementary material for: Evolution of mitochondrial TAT translocases illustrates the loss of bacterial protein transport machines in mitochondria
Source: BMC Biol. 2018 Nov 22;16:141. doi: 10.1186/s12915-018-0607-3 (PMC6251230; doi:10.1186/s12915-018-0607-3)
Supplement: Supplementary file 1 — Figure S1. Protein sequence alignment of eukaryotic TatB. Figure S2. CLANS analysis of TatA from bacteria and mitochondria. Figure S3. Phylogenetic reconstruction of bacterial and mitochondrial TatC. Figure S4. Expression of NgTatC in S. cerevisiae. (DOCX 1194 kb) [file 12915_2018_607_MOESM1_ESM.docx]

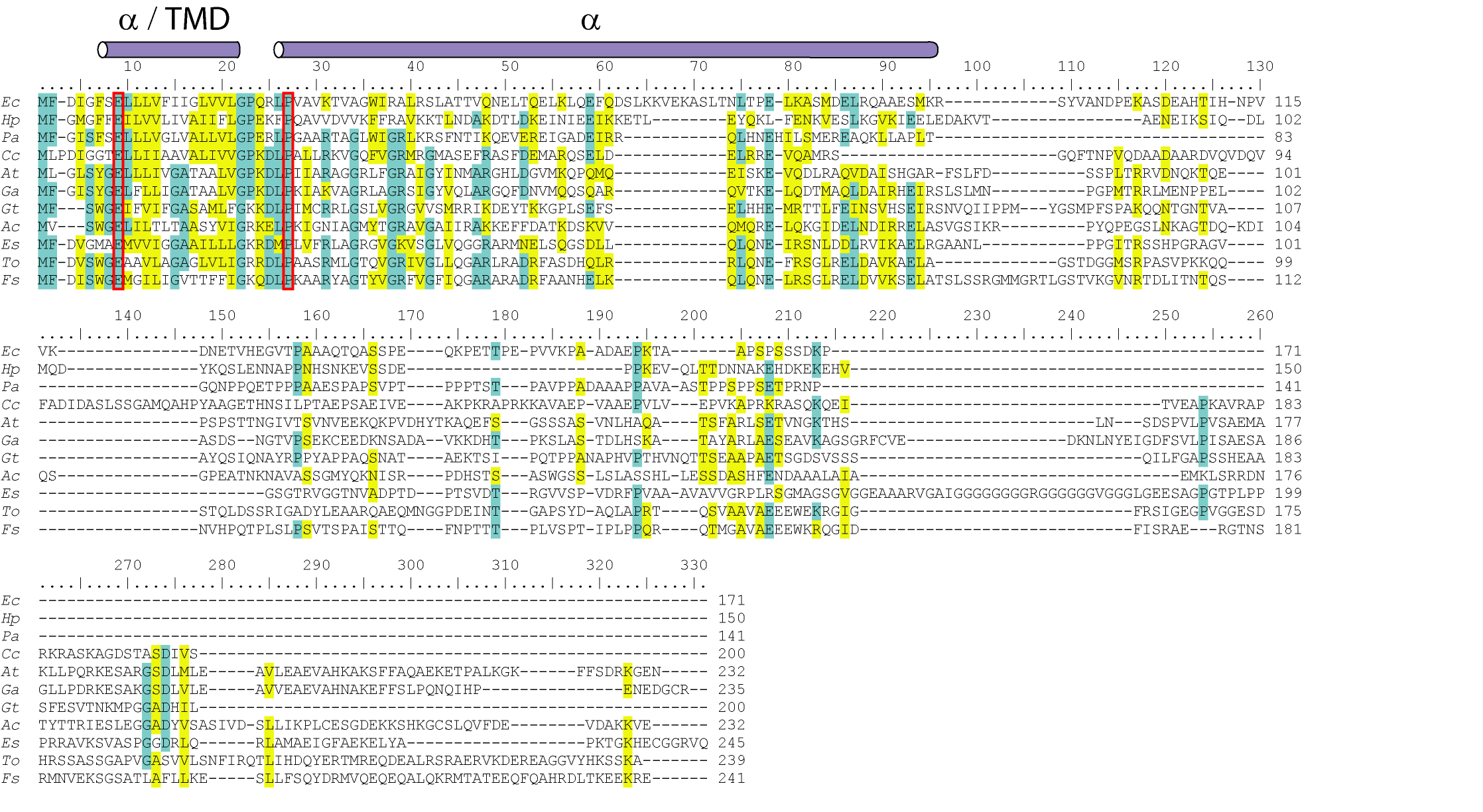


**Figure S1. Protein sequence alignment of eukaryotic TatB.**

Putative mitochondrial TatB sequences were aligned together with the bacterial TatB components using MUSCLE. TatB-specific glutamine and proline residues are highlighted by red rectangles. The violet cylinders represent the transmembrane and the cytoplasm/matrix -exposed α-helices. Ec – *Escherichia coli*, Hp, *Helicobacter pylori*, Pa – *Pseudomonas aeruginosa*, Cc – *Caulobacter crescentus*, At – *Arabidopsis thaliana,* Ga – *Gossypium arboretum*, Gt – *Guillardia theta*, Ac *– Albugo candida*, Es – *Ectocarpus silicosis*, To - *Thalasiossira oceanica*, Fs - *Fistulifera solaris*.


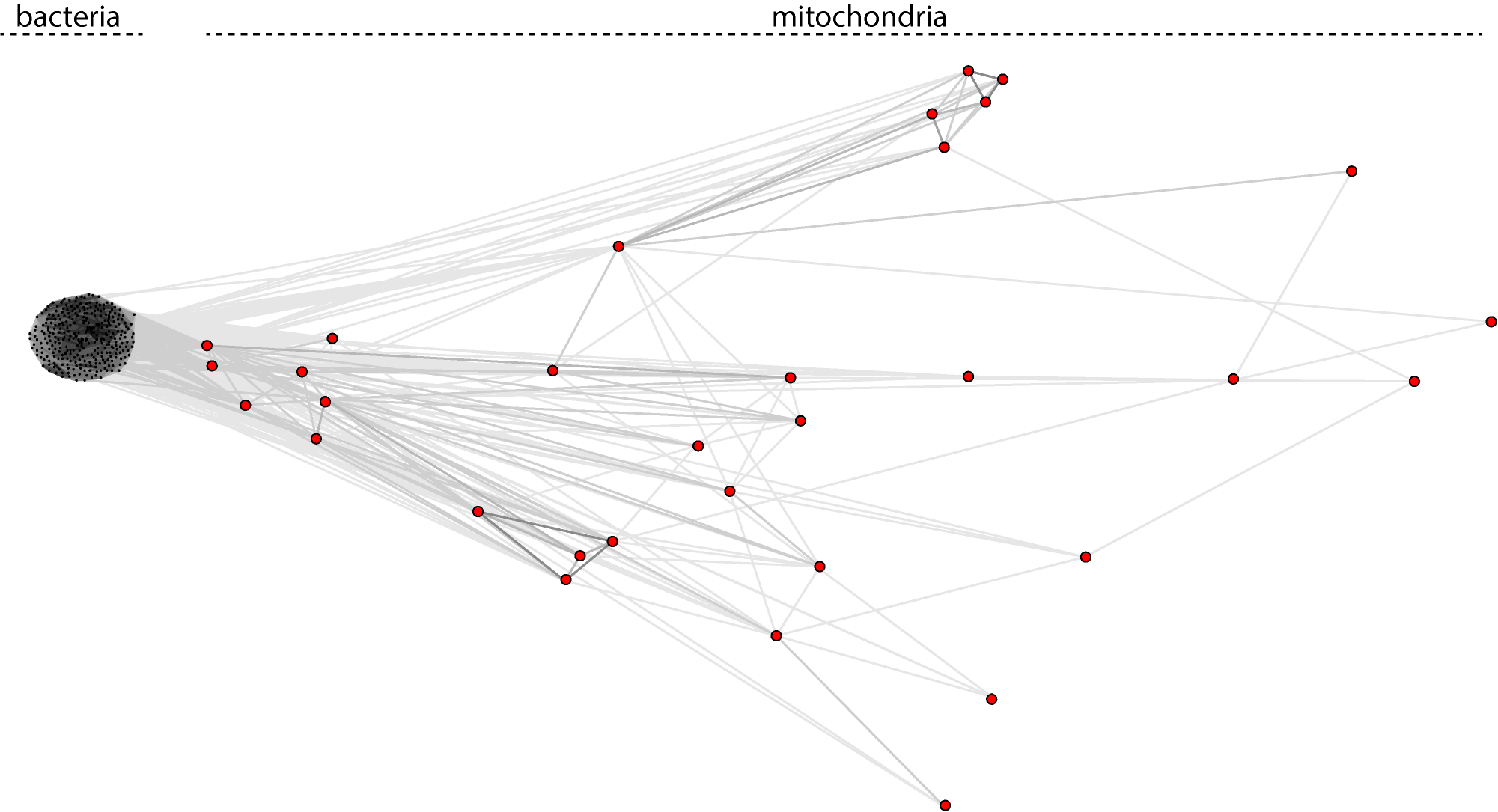


**Figure S2. CLANS analysis of TatA from bacteria and mitochondria.**

Due to the high sequence divergence, the mitochondria-encoded eukaryotic proteins do not form specific cluster as observed for bacterial TatA proteins.

The amino acid sequences of 501 TatA orthologues from bacteria and mitochondria were analyzed by CLANS at https://toolkit.tuebingen.mpg.de. (P value threshold 0.01).


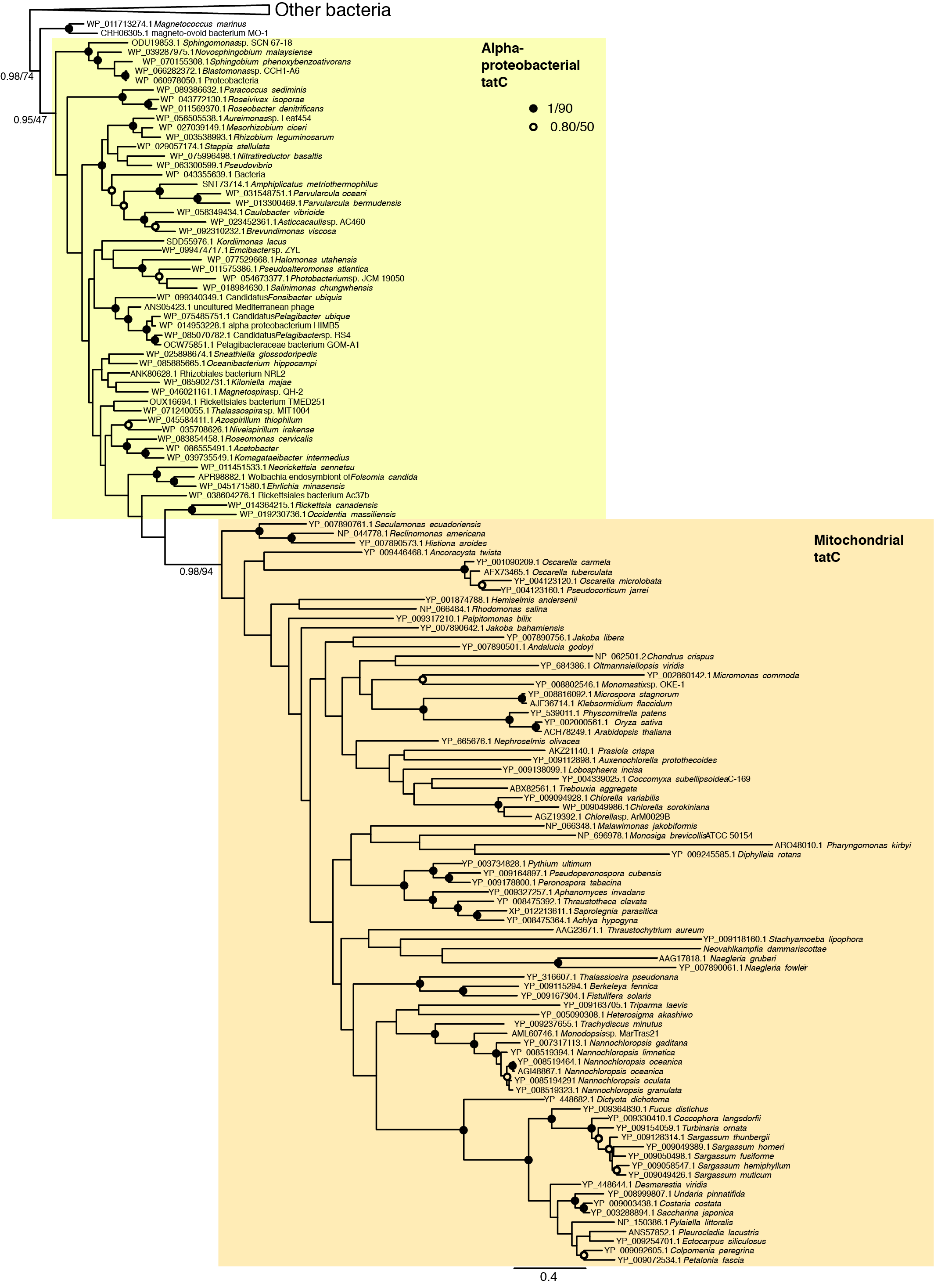


**Figure S3. Phylogenetic reconstruction of bacterial and mitochondrial TatC**. TatC protein sequences encoded in diverse bacterial and mitochondrial genomes were aligned using MUSCLE and subjected to phylogenetic reconstructions using MrBayes for computing posterior probability and RaxML for maximum likelihood. Support values are iconized as inset (MrBayes/RaxML). The MrBayes tree topology is shown.


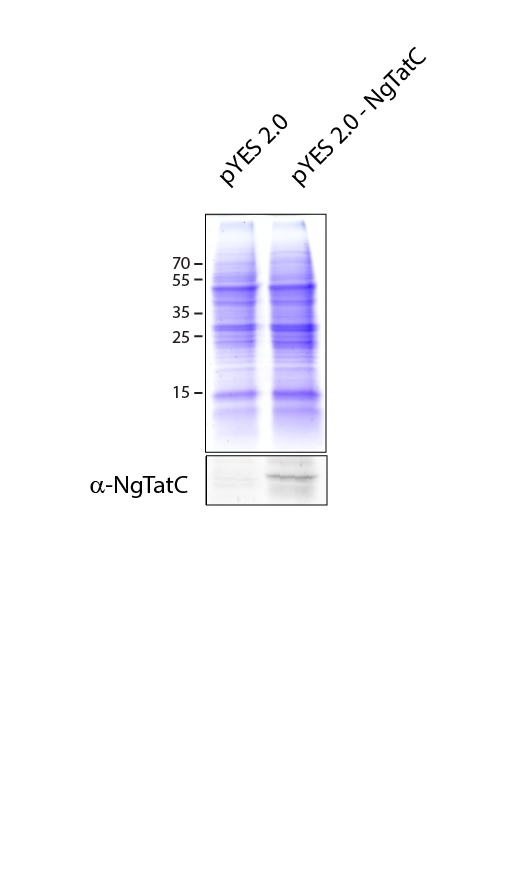


**Figure S4. Expression of *Ng*TatC in *S. cerevisiae*.** Mitochondrial fraction was purified from the yeast transformed with pYES 2.0 carrying *Ng*TatC. The western blot shows the detection of *Ng*TatC only in the mitochondria-enriched fraction isolated from yeast expressing the construct.
